# Enzoötic Abortion in Cows

**Published:** 1885-01

**Authors:** F. S. Billings


### Art. IV.—ENZOÖTIC ABORTION IN COWS.

BY F. S. BILLINGS, V. S.

First, let us understand what we are going to consider.

*Abortion is an accident: it is a result of the action,* in this case of *an unknown something: it is not a disease.* Even pathological changes which might be supposed to anticipate so serious a disturbance do not seem to have attracted much attention, so that we shall have to skip this part of our subject with the remark, that some authors have observed fatty degeneration in the epithelial covering of the placentæ, but have given us no indications leading to what produced it. Fatty degeneration of this nature is a cause of abortion in women, but never of this character. Enzoötic abortion is known to veterinarians and agriculturists, and is essentially a bovine malady, although "Fleming's Obstetrics" calls attention to eruptions of this trouble in other animals and even in women some time in the last century. They are not known to the medical profession of our day, however.

In my paper on Eclampsia, in a recent issue of the JOURNAL I stated the difficulties which surrounded that subject with reference to its nature and causes. Any one who undertakes the study of abortion will find it a still more unsatisfactory task.

So far as knowing anything about it is concerned, the farmer knows as much as the veterinarian, and the latter knows next to nothing. All we know is:

1st. That it occurs.

2d. That the manner of feeding, housing and care, seem to play no essential rôle in originally causing it.

3d. Observation and experience supported by a very few illy controlled experiments, seem to indicate that we have to do with an accident which has for its cause influences of an infectio-contagious nature.

I have said that veterinarians knew very little about it; this is especially true of English and American authors. Williams does not make any mention of it, and it should not be expected of Robertson, as his work is on equine maladies. Steel is most unsatisfactory, and Woodruff Hill is drawn from Fleming who, in his turn, is influenced by the opinions of Continental authors. I know nothing about it myself, and the conclusions which I shall advocate are drawn entirely from the writings of others. It would really seem that very little attention has been given to it in England, if we are to judge from the evidence given in the *London Veterinary Journal*, the leading one of England. It is not indexed from 1875 to date with but one exception, and that a translation of a German article upon the subject. I have taken pains, also, to go through the reports of the Veterinary Associations published in that journal, and have been unable to find any allusion to it; but these things not being indexed I may have passed over some discussions.

As the papers and discussions of these meetings are among the most valuable publications in this journal, we would modestly suggest that their subjects be indexed in the future, as it would greatly increase the value of the publication as a work of reference, which all journals should be.

If we turn to French and German literature we shall not find very much satisfaction, though more has been written upon the subject and more thought given to it: the hypothesis that it is due to infection is well defended and supported by some experimental proofs.

The two best considerations of the subject are of purely American origin, and are to be found in the lengthy report of commissioners appointed by the State of New York to investigate the subject, published in the "Transactions of the New York Board of Agriculture" for the years 1867, '68 and '69, and in the "Report of the Farmers' Meeting of the Massachusetts Board of Agriculture for the year 1883." The first-named authorities do not seem to have arrived at a suspicion of its infectio-contagious origin, as we shall eventually see; while the New England farmers appear to have become more or less convinced of it.

After looking over all these authorities the reader will, like the writer, come to the conclusion that it causes an immense loss to the agriculturist and breeder; and further, that it is the duty of our States to spare no expense in having its causes searched after in the most exact and experimental scientific manner.

Mr. Fleming has called attention to its occurrence earlier than the present century; nevertheless, it did not attract much if any attention until the latter part of the last century. Our New England farmers all speak of it as something belonging to their day and generation, and as an ever increasing evil. It should, I think, be classed with what I have termed "Association or Civilization Diseases," though, as said, not a disease *per se*, that is, it is due to causes which gradually find their origin in the long-continued habitation by animals of the same species, in the same locality, until special unknown conditions are produced which favor, or lead, to the development of specific infectious germs.

That it can cause most serious losses to the cattle interests of our country is most effectually shown by the New York Commissioners' Report.

From various townships they collected statistics of 35,919

cows, reported as having been pregnant: of these 2,057, or .057 aborted.

The percentage of abortions among the cattle of 14 townships was .06. The average number of cattle aborting in each, 22; the average number of affected herds, 29; of non-affected herds, 19.

I would here state that Mr. Sturgis, of the Wyoming Stock Growers' Association, recently wrote me that while, from their manner of raising cattle, it was impossible to gather statistics, still it could not exist to any alarming extent as no complaints had been caused by it among range cattle.

The report alluded to further says with regard to the period of pregnancy at which abortions were noted to have occurred:

| | | |
|---|---|---|
| In the 1st month, | Aborted, | 0 |
| " 2nd " | " | 1 |
| " 3d " | " | 10 |
| " 4th " | " | 66 |
| " 5th " | " | 106 |
| " 6th " | " | 198 |
| " 7th " | " | 341 |
| " 8th " | " | 360 |
| " 9th " | " | 238 |

These figures agree with the observations of the most competent veterinary observers, who look upon the seventh and eighth months as those in which abortion most frequently occurs.

With regard to the time of the year in which the accident is likely to occur we find the following:

| | | |
|---|---|---|
| April, | Abortions, | 86 |
| May, | " | 27 |
| June, | " | 2 |
| July, | " | 2 |
| August, | " | 1 |
| September, | " | 18 |
| October, | " | 40 |
| November, | " | 115 |
| December, | " | 215 |
| January, | " | 354 |
| February, | " | 426 |
| March, | " | 358 |

Such statistics have but little etiological importance; they show, however, that the increase in the number of abortions bears a direct relation to the period at which most cattlemen desire to have their cows "come in"; that is, to be ready for the spring grasses. They show further that some general cause must be at work at such times other than the manner of feeding, etc., for if ergot, as has been assumed, were the cause, the cows would be as liable to gain access to it in November and December, as in January, February, and March.

The New York Commissioners also gave considerable attention to the influence, if any, exerted by the age of the bull used to serve the cows, and report:

| No. Cows impregnated by | Bull 1 year old. | Bull 2 years old. | Bull 3 years old and over. |
|---|---|---|---|
| Carried to full term, . . | 9,740 | 19,530 | 1,482 |
| Aborted, . . . . . | 522 | 1,340 | 74 |
| Per cent. of abortions, . . | .05 | .064 | .047 |

which gives a slight advantage for the older bulls.

In the "*Mittheilungen aus der Praxis*," in Prussia, Fischbach reports a case of two bulls, one three-and-a-half years old, the other, two. Some forty cows were served by the older bull, all of which aborted between the sixth and thirteenth week of pregnancy, while the accident did not occur in any of those served by the younger. The most exact examination did not reveal any reason therefor in the bull.

Roloff reports a case in which *all* the cows in one stable aborted, while of those in another on the same farm none aborted. Care, feed, ventilation, etc., were exactly alike in each stable.

In another stable where traffic caused an almost constant change in the animals, which were of every variety of breed, and gathered from all parts of Germany, nearly every pregnant cow aborted one after another.

In another, abortion still occurred after every possible change in the manner of feeding.

Abortion has been frequently observed to occur in cows fed upon brewery and distillery refuse, in Germany, by Richter, who claims that, looking upon an insufficiency of salt in such feed as the cause, it entirely ceased when this failure was made up by the addition of the proper quantity of saline ingredients to the food. It is only necessary to say that this is not the kind of abortion we are considering. Roloff also reports cases in which he has observed the trouble existing in stables for a number of years, constantly increasing until, finally, nearly every pregnant cow aborted, which shows that the cause had become domiciled.

In a stable on a farm in Saxony one cow after another aborted while among those in another on the same farm none occurred though they all had the same care, but upon a maid assisting at an abortion in the first stable and then going back among the cows in the second stable, to which she regularly attended, abortion set in among them and continued for a long time.

These examples, particularly the last one, are sufficient to indicate that there must be some special specific cause for this trouble which we should consider, and that this is of an infectious or contagious nature.

It must be remembered that, while all contagious diseases are of an infectious type, still all so called infectious maladies are not necessarily of a contagious nature.

### ETIOLOGY.

I have already said that the New York Commissioners did not seem to have had any idea that the cause of this disturbance was an inficiens, hence it is but just to them, and in accordance with our present purpose, to let them speak for themselves. They appear to have come to the conclusion that abortion was due to unnatural conditions produced in cows from the inconsiderate forcing process to which man's greed puts the sexual and milk producing organs of cattle, both with reference to degree and also to the unnaturally early age at which we cause these functions to be called into full activity.

After describing the manner of "selection" by which man has gradually produced our present milk machines, they tell us that "if, however, instead of following these rules, the attempt be made to get a large increase at once; first, by forcing the uterine reproductive apparatus into activity before the animal has arrived at full maturity, and afterwards by continuing the drain upon the mammary secretion at the same time that a second fœtus is getting its supply from the placenta: by the first practice the uterine reproductive apparatus is weakened, and a liability to abortion established; and by the second, the natural supply of blood, which should go, during pregnancy, to the uterus to nourish the fœtus, is continued to be drawn in the other direction towards the mammary gland, arrest of development from inanition is endangered, and when it occurs the fœtus is expelled as a foreign body." It is scarcely necessary to say that such induced conditions do not have, for themselves, any self-evident characteristics to lead us to consider them as playing any essential part in the production of the form of abortion we are considering.

Even this report frequently quotes cases of abortion appearing in herds on the introduction of a cow from a farm on which it had prevailed, but this fact does not seem to have made any impression on these investigators.

Though the causes inferred by the New York Commission are very suggestive in many ways of the evils to which the greed of breeders subjects the bovine family, still they do not coincide with the generally accepted views of to-day.

The New York Commmissioners sum up their work as follows:

"The affirmative results obtained are :

"1st. That cows which have first calved at or under three years of age, are more liable to abort during their subsequent pregnancies than those that first calve at three years of age or over, in the proportion of 5 to 3.

"2d. That cows subjected to removals at any time, are liable to abort over those raised on the farms, in the proportion of 7 to 4½."

(This requires close observation.)

"3d. That cows subjected to removals during pregnancy, are liable to abort over those not moved at such times in the proportion of 9 to 2."

"4th. That arrest of development is the condition immediately preceding abortion."

In support of this they tell us that "of 4163 abortions in the year 1867-8, 3597, or 86 per cent., of the young were reported as 'dead' at the time they were aborted."

Still another cause has been considered by many to play a very essential rôle in the production of enzoötic bovine abortion, viz., ergot. The conditions produced are known as

ERGOTISM, of which we heard so much during the spring of 1884, from Kansas.

For a very exact and detailed account of the invasion of grains and grasses by this parasite, we must again refer the reader to the New York Report, for to do it here would make our paper too long for a journal article, and we object to continued papers when they can be avoided.

The great value of the work of the New York Commissioners is that they have well prepared the ground for further investigation by showing that many things which have been assumed to have causal connection with bovine abortion do not have any, at least with its enzoötic form.

With reference to plants liable to cause it, they say "that they have been unable to find any plant, in itself healthy, of which there is a reasonable probability of its being the cause of this trouble, affecting as it does so large a number of farms."

"Ergot was found on ten different plants, of which six are among the more common meadow or pasture grasses."

No positive connection could be discovered between the existence of ergotized grasses and abortion.

Some cases have been reported in veterinary journals which, if taken by themselves, would seem to indicate that ergot can cause abortion in cattle.

Heusinger (*München Zeitschrift, vol. ii, p.* 101) says: "Ergot that is gathered with grains or grasses in an extremely hot and dry harvest season not only falls out easily but is of a very pale color, and inactive; while in wet summers the ergot is black, not easily detached, and possesses its active properties to the fullest degree."

He again quotes from "*The Academy*," an English publication, of January 9th, 1875, "that in New Zealand an outbreak of enzoötic ergotism was observed in cattle and sheep. It was not of a fatal character, but interfered greatly with the productivity in young of the animals. It occurred in the fall; the animals were lame and had convulsions. It ceased upon changing them to other pastures."

Hazelbach, G. & H., xxvi, p. 211, reports an interesting case of abortion in cows. He was called in consultation by a farmer who reported that eleven of his best cows had recently aborted without any known cause. The cows all appeared healthy, aside from the consequence of this accident. Their food was wheat from the field and straw. On going to the field a large quantity of rust—*Ustilago Moidis*—was found upon the wheat, of which H. gathered quite a quantity.

He provided two pregnant bitches, one of which was due to whelp in three and a half, the other in one and a half weeks. He gave them one-half ounces powdered, of this fungus; and the next day, two drachms; violent pains soon developed after the second dose, and in two hours abortion occurred in the bitch due in three and a half weeks, and soon after in the other. No further cases occurred in the cows when the wheat was taken from them.

We have already alluded to the singular outbreak in Kansas, which one veterinarian declared positively to have been "Contagious Foot and Mouth Disease," an opinion I believe he still adheres to, while two others declared it to be "Ergotism," and a third affirmed it to be some form of "Foot-rot," an indefinite term, for ergotism can be accompanied by necrosis in the extremities.

We admit our inability to come to any definite conclusion on this testimony, as we have it in the *American Veterinary Review*, but are firmly convinced that it was not the "Foot and Mouth Disease," while from our appreciation of Dr. Salmon's ability, we feel inclined to adopt his opinions, supported as they are by the later published observations of Dr. Law. While each attempted to prove the other wrong in his diagnosis, it is much to be regretted that no exact and detailed account of the symptoms, at least of a positively satisfactory nature, can be drawn from these reports.

It is said "that the bodily temperature was but little augmented; that the appetite remained good throughout, and that there was no trouble in masticating or swallowing."

Another says "the animals stopped eating grain, but ate hay; seemed very stiff; frothed at the mouth, and appeared in great agony. Coldness of the extremities, with necrosis of the whole or parts of the lower portion, was not uncommon."

Another says "the mouth was hot and reddened. Three blisters were to be seen on its roof, and two on the tongue"—of one animal.

Still another tells us "that he does not consider the trouble to have been due to ergotized rye, but rather to have been the 'foot-rot,' which is quite common at times." In proof of this conclusion, he says that "on Mr. G's. farm, where the disease prevails, there is no ergotized rye in the hay; but stranger still, *Mr. B. has fed seventy-five cattle all winter on hay that is full of ergot and only three heifers and one cow are affected;* another puzzle is presented by Mr. K's buying sixty-three young cattle of Mr. D. on the 15th of December, '83, and on the 23d nearly all were down with the disease. K's hay contained ergotized rye, while Mr. D. had no sickness in his herd."

This last authority, Dr. Hopkins, quotes Finlay Dun as saying: "it—ergot—does not, however, as in man, cause gangrene in the extremities." Dun (p. 294), quoting Dr. Wright, says: "Gangrene of the extremities is not, however, produced so readily as in man," which is not saying that "it does not cause gangrene." Again Dun says: "Mules fed on it—ergot—lose their hoofs, and fowls lay eggs without shells." It is especially to be noticed, that even those veterinarians that looked upon the Kansas trouble as ergotism, do not record a single case of its having been characterized by abortion, or even a suspicion of it.

In this regard, Dun says (p. 295): "Concerning the power of ergot to induce contractions of the uterus and expulsion of its contents, there is still much difference of opinion. Amongst the lower animals it certainly has no power of producing abortions. It has been given in large and repeated doses to cows, bitches, cats, swine and rabbits in all stages of pregnancy, but without causing abortion."

With regard to its constitutional effects, he says: "when given for several weeks it caused, in dogs and rabbits, nausea, impaired appetite—the Kansas cows ate well—a weak and irregular pulse, diarrhœa, excessive fœtor of the secretions and excretions, paralysis, particularly of the posterior extremities enlargement of the limbs, contraction of the spleen, tubercles in the lungs and mesentery—? B.—impairment of all the senses, wasting and general debility."

V. Boeck—*Handbuch der Intoxicationen—Ziemssin*, (vol. 15, p. 576), says, with reference to its abortine action in woman: "the so frequently affirmed and denied influence of ergot upon the movements of the uterus, require but brief mention, as change of this organ in acute ergotism do not belong to the essential phenomena. Obstetricians have confirmed the fact that ergot can increase the intensity of the pains in pregnant woman, but many deny that it can cause the uterine contractions of itself." Schlesinger and Wernich, however, report having experimentally caused such contractions in the uterus of animals. Some veterinary authors deny this action. This action is not, however, the primary, but rather the secondary action of ergot. It is readily to be seen that ergot, even in large quantities, is not considered to be by any means a direct and sure abortivum. In this regard, and also with reference to the somewhat questionable character of the Kansas trouble, it would seem that our government should endeavor to procure a large quantity of ergotized grain—the ergot being right in quality, and carry on a long continued series of feeding experiments, under the supervision of an exact and competent veterinarian, upon cattle, and in order to test two questions at once, the cattle to be selected should be cows in the earlier months of pregnancy.

We come now to the consideration of what seems to be the cause of enzoötic abortion in cows, viz.: An Unknown Quantity.

### AN INFICIENS.

We have already quoted quite a number of practical observations in support of this theory, and have stated that the most intelligent among our New England farmers have also come to the conclusion that the trouble is not only infectious, but also contagious.

As illustrating this point, Stockfleth—a Danish veterinarian—reports a case where eleven out of fifty-five cows aborted in one day. Also of one cow that aborted three times in one year.

Dr. Salmon (Mass. report) speaks as if there were grounds for believing that after the third abortion a cow might be expected to carry future calves to full term.

While we think we know his reasons for such an assertion, we have been unable to find any sufficient evidence for the same, and think it a question of significant importance, only to be settled by exact statistical observations. Farmers seldom keep a given cow that has aborted, long enough to enable us to satisfactorily answer such a question.

Brauer, a German veterinarian, reports that the trouble ceased in a stable after causing it to be carefully cleaned and disinfected. Dr. Salmon reports a similar experience, and one of my own patrons reports like results. Brauer caused abortion to occur in a gravid cow in nine days, after introducing into her vagina some of the lochial discharges from a cow that had just aborted. In another case, he saw abortion follow the same procedure in cows after the elapse of 12, 15, 21, 11 and 15 days.

He considers some unknown micro-organism to be the cause.

Lehnhert looks upon it as due to infection when it runs through a stable of cows.

He speaks most favorably of the results of cleansing and disinfecting. He produced it in two cows in the 7th month of pregnancy, in eighteen and twenty days by introducing the lochial discharge from aborted cows into the vagina.

He recommends the speediest possible removal of the afterbirth from the aborted cows, with the thorough clensing and disinfection of the birth passages, and the immediate removal of all gravid cows from a stable in which an abortion has occured. Weigel reports a wide spread enzoötic of this trouble which extended over six large farms.

Roloff thinks it to be due to some infectious miasma which enters, *per vaginam*, into the uterus. He has frequently seen the vulva and vagina injected and swollen antecedent to the accident. Disinfection and the removal of gravid cows is generally followed by favorable results.

Franck has similar views, which he has supported by experiments of the same nature as those of Brauer, with like results. He makes a seeminglybro ad statement when he says "that ichorous septic material, when brought into contact with the sexual organs, can cause abortion." We know that he means the lochial discharge from aborted cows, however.

He claims to have seen or found in the vagina of such cows a development of leptothrix bacteria upon the mucosa, which gradually increased in quantity until abortion occurred.

This is the evidence. What does it suggest?

1. It shows the value of close observation and of the application of general principles to conditions of which we know very little about.

Experience, experiment and observation have thus taught us that although we do not know the cause of enzoötic abortion in cows, still that we can prevent its further extension in a given herd by the timely cleansing and disinfection of the stable, and the isolation and removal of the remaining pregnant cows to other quarters.

Large places should have a sufficient number of "lying-in boxes" to which cows should be removed in time. After use, they should be scalded out, white-washed and disinfected, exposed to the air daily, and never used except for calving cows. When not in use they should have bare walls and floor. No litter, straw or stable utensils should be left in them. Before use they should be once more disinfected, freshly littered by persons with other boots and clothes on than used in general about the cattle in the main stable.

Such a procedure will put an end to the extension of bovine abortion, if we never find the cause. No enzoötic outbreak need ever trouble the large cow-owners if this treatment is adhered to.

2. That the isolation of a specific, abortion-producing germ is a matter of the greatest difficulty on account of the ill-apposition of the vulvo-vaginal walls and the consequent presence of almost innumerable numbers of many varieties of micro-organisms at times, and in cows when no abortion is occurring, and even when not pregnant.

3. Can other septic materials, such as putrifying meat, mixed with water, which is full of germs that cannot be distinguished from many of the above-mentioned forms, also cause abortion in cows if injected into the vagina?

4. Can lochial discharges from aborted cows—assuming that they can produce it in cows—also produce abortion in other gravid animals—mares, sows, dogs, etc.?

5. What is the condition of the *os uteri* during the various stages of pregnancy?

6. What is the condition of the blood, liver, spleen, etc., of such cows with reference to germ infection?

7. Experiments should be made with the lochial discharges of aborted cows upon the more rugged native animals, as well as upon the higher grade animals generally held by butter and milk producers at the present time.

8. Statistics of every conceivable variety should be gathered by competent men.

9. These questions can only be settled by the State, which should supply all the money to carry on the necessary researches.

10. But little can be learned from fanatical observations alone. Experiment is the only method which will lead to satisfactory results.

11. None but the most scientifically qualified veterinarians are suited to such work. Mere practical men, no matter what reputation they may enjoy, wont do.

